# Sub-clinical mastitis and associated risk factors on lactating cows in the Savannah Region of Nigeria

**DOI:** 10.1186/1746-6148-8-134

**Published:** 2012-08-15

**Authors:** Aminu Shittu, Jamilu Abdullahi, Aliyu Jibril, Aminu A Mohammed, Folorunso O Fasina

**Affiliations:** 1 LUCINDA Research Group, Department of Epidemiology and Population Health, Institute of Infection and Global Health, University of Liverpool, Leahurst Campus, Chester High Road, Neston, Cheshire, CH64 7TE, United Kingdom; 2 Department of Theriogenology and Animal Production, Faculty of Veterinary Medicine, Usmanu Danfodiyo University, P.M.B 2254, Sokoto, Nigeria; 3 Department of Parasitology and Entomology, Faculty of Veterinary Medicine, Usmanu Danfodiyo University, P.M.B 2254, Sokoto, Nigeria; 4 Department of Production Animal Studies, University of Pretoria, Onderstepoort, 0110, South Africa

**Keywords:** Risk factors, Sub-clinical mastitis, Cattle, Nigeria

## Abstract

**Background:**

Sub-clinical mastitis limits milk production and represents an important barrier to profitable livestock economics worldwide. Milk production from cows in Nigeria is not at optimum levels in view of many factors including sub-clinical mastitis.

**Results:**

The overall herd-level prevalence rate for SCM was 85.33% (256/300 heads of cows) while the quarter-level prevalence rate of SCM was 43.25% (519/1,200 quarters). The prevalence of SCM was 50.67%, 43.67%, 39.67% and 39.13% for the left fore-quarter, right hind-quarter, left hind-quarter and right fore-quarter, respectively. The Rahaji breed had the highest prevalence of SCM with 65.91% (29/44), while the White Fulani breed had the least with 32.39% (57/176). A total of 32.33% (97/300) had only one mammary quarter affected, 30.33% (91/300) had two quarters affected, 16.00% (48/300) had three quarters affected while 6.67% (20/300) had all the four quarters affected. A total of 53.00% had SCM in multiple quarters (159/300). The risk of SCM decreased significantly among young lactating cows compared to older animals (OR = 0.283; *P* < 0.001; 95%CI = 0.155; 0.516). The Rahaji breed had significantly higher risk compared with the White Fulani breed (OR = 8.205; *P* = 0.013; 95% CI = 1.557; 43.226). Improved sanitation (washing hands before milking) will decrease the risk of SCM (OR = 0.173; *P* = 0.003; 95% CI = 0.054; 0.554).

**Conclusion:**

SCM is prevalent among lactating cows in the Nigerian Savannah; and this is associated with both animal characteristics (age, breed and individual milk quarters) and milking practices (hand washing).Good knowledge of the environment and careful management of the identified risk factors with improved sanitation should assist farm managers and veterinarians in implementing preventative programmes to reduce the incidence of SCM.

## Background

Sub-clinical mastitis (SCM) remains an economically important condition for the dairy industry [1]. SCM is one of the major causes of animal suffering, responsible for poor growth in young animals, reduced milk quality, poor product hygiene and undesirable changes in the milk’s composition [2-5]. Mastitis is characterized by physical, chemical and bacteriological changes in the milk and pathological changes in the glandular tissue of the udder [6-8]. Effective mastitis control strategies depend on early and accurate detection, since proactive management of the condition can reduce the negative effects of the disease and achieve higher cure rates [9,10].

Previous studies on mastitis in livestock in Nigeria have been focused primarily on small ruminants which supply the lesser percentages of the national milk requirements. To date, no published reports have examined the condition in cattle or evaluated the risk factors for SCM in lactating cows [11-15]. However, several cases of mastitis have been recorded in cows presenting at the Veterinary Teaching Hospital, Usmanu Danfodiyo University, Sokoto, Nigeria and other such locations in Nigeria, it is therefore important to assess the situation and recommend preventative measures.

In this study, California mastitis test (CMT) was used to determine the prevalence of quarter and herd-level sub-clinical mastitis for individual cows and the herds. This study also investigated potential risk factors associated with QSCM. The outcome is expected to enhance better planning of a mastitis control programme with implications for the Nigerian dairy industry. It also provides strong evidence for the value of hand washing, a message that the extension veterinarians can pass to farmers to reduce the risk of SCM.

## Results

### Descriptive results

Five hundred and nineteen (43.25%) milk samples from the four mammary quarters were CMT positive, while 681 (56.75%) samples were CMT negative (Table 1). The individual quarter-level prevalence of SCM was 50.67%, 43.67%, 39.67% and 39.13% for the left fore-quarter (LFQ), right hind-quarter (RHQ), left hind-quarter (LHQ) and right fore-quarter (RFQ), respectively. Among the 4 breeds of cattle included in this study, Rahaji breed had the highest prevalence of SCM with 65.91% (29/44), Sokoto Gudali had 45.67% (190/416), Holstein Friesian had 43.09% (243/564) and the White Fulani breed had the lowest with 32.39% (57/176) (Table 2). On the herd level, a total of 32.33% (97/300 heads of cows) had only one mammary quarter affected, 30.33% (91/300) had two quarters affected, 16.00% (48/300) had three quarters affected while 6.67% (20/300) had all the four quarters affected. Thus a total of 53.00% had SCM in multiple quarters (159/300). The overall herd-level prevalence was 85.33% (256/300 heads of cows) and quarter level prevalence was 43.25% (519/1200 quarters) (Table 2). Based on the intensity of positivity, a majority, 310/519 (59.73%) of the positive samples were weakly positive, while 170/519 (32.76%) were moderately positive and 39/519 (7.51%) were strongly positive (Table 3).

**Table 1:** Descriptive data showing the prevalence of SCM at quarter level

**Quarter**	**SCM**		
	**Positive (%)**	**Negative (%)**	**Total (%)**
**LFQ**	152 (50.67)	148 (49.33)	300 (100.00)
**LHQ**	119 (39.67)	181 (60.33)	300 (100.00)
**RFQ**	117 (39.13)	183 (60.87)	300 (100.00)
**RHQ**	131 (43.67)	169 (56.33)	300 (100.00)
**Total**	519 (43.25)	681 (56.75)	1200 (100.00)

SCM = sub-clinical mastitis; LFQ = Left fore-quarter; LHQ = Left hind quarter; RFQ = Right fore-quarter; RHQ = Right hind quarter.

**Table 2:** Descriptive data showing prevalence of QSCM by cow breed

**Breed**								
	**White Fulani**		**Sokoto Gudali**		**Holstein Friesian**		**Rahaji**	
**Quarter**	**SCM (%)**	**Total (%)**	**SCM (%)**	**Total (%)**	**SCM (%)**	**Total (%)**	**SCM (%)**	**Total (%)**
**LFQ**	17 (38.64)	44 (100.00)	57 (54.81)	104 (100.00)	71 (50.00)	141 (100.00)	7 (63.64)	11 (100.00)
**LHQ**	15 (34.09)	44 (100.00)	41 (39.42)	104 (100.00)	55 (39.01)	141 (100.00)	8 (72.73)	11 (100.00)
**RFQ**	12 (27.27)	44 (100.00)	47 (45.19)	104 (100.00)	51 (36.43)	141 (100.00)	7 (63.64)	11 (100.00)
**RHQ**	13 (29.55)	44 (100.00)	45 (43.27)	104 (100.00)	66 (46.81)	141 (100.00)	7 (63.64)	11 (100.00)
**Total**	**57 (32.39)**	176 (100.00)	**190 (45.67)**	416 (100.00)	**243 (43.09)**	564 (100.00)	**29 (65.91)**	44 (100.00)

QSCM = sub-clinical mastitis; LFQ = Left fore-quarter; LHQ = Left hind quarter; RFQ = Right fore-quarter; RHQ = Right hind quarter.

**Table 3:** Results of four categories representing CMT scores by cow breed

**Breed**	**Intensity of infection**				
	**Negative (%)**	**Weak positive (%)**	**Positive (%)**	**Strong positive (%)**	**Total (%)**
**White Fulani**	119 (67.61)	37 (21.02)	16 (9.09) 16 (9.09)	4 (2.27)	176 (100.00)
**Sokoto Gudali**	226 (54.33)	124 (29.81)	53 (12.74)	13 (3.13)	416 (100.00)
**Holstein Friesian**	321 (56.91)	133 (23.58)	92 (16.31)	18 (3.19)	564 (100.00)
**Rahaji**	15 (34.09)	16 (36.36)	9 (20.45)	4 (9.09)	44 (100.00)
**Total**	681 (56.75)	310 (25.83)	170 (14.17)	39 (3.25)	1200 (100.00)

CMT = California Mastitis Test; – (negative; SCC score of ≤100,000 cells/ml); + (weak positive; SCC score of >100,000 – 500,000 cells/ml); ++ (positive; SCC score of >500,000 – 1,000,000 cells/ml) and +++ (strong positive; SCC score of ≥1,000,000 cells/ml). SCC = somatic cell count.

### Risk factors associated with CMT - positive quarters and SCM

The 10 herd management-associated risk factors that were dropped from the analyses due to many outliers and wide disparities included the question numbers 6, 8, 9, 12, 13, 18, 19 – 21 (see Table 4).

**Table 4:** Univariable analysis of risk factors associated with SCM in lactating cow on herds in the Savannah region of Nigeria

**Variable**	**OR (crude)**	**SE**	**z**	***P*value**	**95 % CI**
Quarter	0.918	0.048	−1.65	0.100	0.828; 1.016
Month	1.223	0.098	2.52	0.012	1.046; 1.430
Herd size	0.910	0.002	−0.31	0.758	0.996; 1.003
Management system	1.254	0.150	1.89	0.059	0.992; 1.585
Breed	1.241	0.094	2.85	0.004	1.069; 1.439
Cow type	1.333	0.159	2.40	0.016	1.054; 1.685
Cow origin	0.987	0.115	−0.11	0.914	0.785; 1.241
Age (years)	2.041	0.243	5.99	<0.001	1.616; 2.577
Parity	2.081	0.271	5.62	<0.001	1.611; 2.686
BCS	0.999	0.069	−0.01	0.991	0.873; 1.144
Washing of teat	0.755	0.100	−2.11	0.035	0.582; 0.980
Pre-striping before milking	0.359	0.155	−2.37	0.018	0.154; 0.836
Washing hands before milking	0.314	0.085	−4.28	<0.001	0.184; 0.533
Heifer and cow	0.245	0.121	−2.86	0.004	0.093; 0.643
Replacement heifers	0.926	0.130	−0.55	0.584	0.703; 1.219
Feeding after milking	2.787	1.203	2.37	0.018	1.196; 6.496

^*^Associations were evaluated by obtaining a crude OR estimate; variables with values of *P* ≤ 0.20 were considered significant and included in subsequent multivariable analysis; SCM = sub-clinical mastitis.

The remaining 12 cow and herd-level explanatory variables were found to be significantly associated with SCM at *P* ≤ 0.20, based on a CMT-positive quarter (Table 5). In the correlation analysis (Table 6), age and parity were strongly correlated (*r* = 0.96).Age was considered the more relevant variable based on biological plausibility, thus parity was dropped from the model. In the LRT, 3 variables (cow type, management system and feeding of cows after milking) were automatically dropped in the initial model-building due to multicollinearity. The final model, the multivariable mixed-effects logistic regression model to examine the effect of potential risk factors on prevalence of SCM, included age of cow in years, washing of hands before milking, breed of cow, and quarter. Young lactating cows were less likely to develop SCM than older lactating cows (OR = 0.283; *P* < 0.001; 95%CI = 0.155; 0.516). Cows in herds where hand-washing before milking is being practiced frequently had a reduced risk of developing SCM than cows in herds where hand washing is not being observed (OR = 0.173; *P* = 0.003; 95% CI = 0.054; 0.554). The Rahaji breed is more likely to develop SCM compared with the White Fulani breed (OR = 8.205; *P* = 0.013; 95% CI = 1.557; 43.226). Holstein-Friesian breed, though less likely to develop SCM compared to Rahaji similarly have a higher tendency compared with White Fulani (OR = 1.914; *P* = 0.156; 95%CI = 0.781; 4.688). Sokoto Gudali is also more likely to develop SCM than White Fulani (OR = 2.601; *P* = 0.042; 95%CI = 1.036; 6.533) (Table 7). At the individual quarter level, the LFQ is more likely to develop SCM than the other quarters.

**Table 5:** Correlation matrix for the broad set of explanatory variables considered in the analysis of risk factors associated with the SCM among lactating cow in the Savannah region of Nigeria

**Variable**	**A**	**B**	**C**	**D**	**E**	**F**	**G**	**H**	**I**	**J**	**K**	**L**
**A**	1											
**B**	0.9637	1										
**C**	0.0273	0.0148	1									
**D**	0.143	0.0945	0.0702	1								
**E**	0.0397	0.0434	0.5789	0.0182	1							
**F**	−0.1778	−0.1315	−0.3027	−0.2847	−0.1752	1						
**G**	−0.0949	−0.0563	−0.0903	−0.208	−0.1284	0.3426	1					
**H**	0.018	0.0298	0.5884	0.022	−0.027	−0.1781	0.0397	1				
**I**	−0.018	−0.0298	−0.5884	−0.022	0.027	0.1781	−0.0397	−1	1			
**J**	−0.019	−0.0751	0.4626	0.1916	0.2678	−0.6544	−0.217	0.2722	−0.2722	1		
**K**	−0.1964	−0.1503	−0.3469	−0.3396	−0.2008	0.8725	0.373	−0.2041	0.2041	−0.75	1	
**L**	−0.0015	−0.0016	0.0004	−0.0012	0.0073	0.0016	0.0012	0.0002	−0.0002	0.0009	0.0018	1

A = age, B = parity, C = washing hands before milking, D = breed, E = heifer and cow, F = month, G = cow type, H = pre-striping before milking, I = feeding after milking, J = washing of teats, K = management system, L = quarter.

**Table 6:** Final multivariable mixed-effects logistic regression model, with cow nested in herd being random factors, of risk factors significantly associated with SCM in the Savannah region of Nigeria

**Variable**	**Level**	**OR (adjusted)**	**SE**	**z**	***P*value**	**95%CI**	^*^Chi square	^*^DF	^*^Pvalue
Age (years)	Old	1.00	Ref	Ref	Ref	Ref	17.40	1	< 0.001
	Young	0.283	0.087	−4.11	< 0.001	0.155; 0.516			
Washing hands before milking	No	1.00	Ref	Ref	Ref	Ref	9.05	1	0.003
	Yes	0.173	0.103	−2.95	0.003	0.054; 0.554			
Breed	White Fulani	1.00	Ref	Ref	Ref	Ref	7.83	3	0.050
	Sokoto Gudali	2.601	1.222	2.03	0.042	1.036; 6.533			
	Holstein-Friesian	1.914	0.875	1.42	0.156	0.781; 4.688			
	Rahaji	8.205	6.956	2.48	0.013	1.557; 43.226			
Quarter	LFQ	1.00	Ref	Ref	Ref	Ref	17.13	3	0.001
	LHQ	0.462	0.103	−3.46	0.001	0.298; 0.715			
	RFQ	0.446	0.010	−3.61	< 0.001	0.287; 0.691			
	RHQ	0.614	0.135	−2.22	0.027	0.399; 0.945			

^*^ The effect of potential risk factors in the multivariable mixed-effects logistic regression model; SCM = subclinical mastitis.

**Table 7:** Summary of questions in relation to improved udder health and analysed for association with SCM in lactating cow on herds in the Savannah region of Nigeria

**S/No.**	**Question**	**Response type**
1.	Herd identification	Numeric
2.	Herd size	Numeric
3.	Do you wash hands and/or wear gloves during milking?	Yes/No
4.	Do you pre strip as part of your preparation before the cows are milked?	Yes/No
5.	Do you wash the teats as part of your preparation before milking?	Never/ Only the cows with dirty udders/all cows
6.	Do you use paper towels for the preparation of the udder before milking?	Yes/No
7.	Do you use a (wet) cloth for preparation of the udder?	Yes/No
8.	Do you use teat dipping/spraying before milking?	Yes/No
9.	Do you use teat dipping/spraying after milking?	Yes/No
10.	How regularly are your milking equipments washed?	Daily/weekly/monthly
11.	How regularly are your milking equipments replaced?	Monthly/annually
12.	Which of the following options describe your dry cow therapy best?	No dry cow therapy used/ Selective in the cows which I used dry cow therapy on/ I use dry cow therapy on each cows which is dried off
13.	Do you have a separate calving paddock for your cows?	Yes/No
14.	Do heifers and cows have the same calving paddock?	Yes/No
15.	Do you buy replacement heifers?	Yes/No
16.	Do you feed your cows after milking?	Yes/No
17.	If yes**,** are they fed in a feed pad or in a paddock? If not continue with question 17	Feed pad/paddock
18.	Do you feed additional supplement to your lactating cows, choose one of the following option	To all lactating and dry cows/only lactating cows/only dry cows/other options like
19.	Is mastitis a primary reason for you to cull a cow?	Yes/No
20.	Do you check the udder health of individual cow timely?	Yes/No
21.	Do you record treatments of clinical cases of mastitis?	Yes/No

## Discussion

This study indicates that there is an association between certain risk factors and SCM in lactating cows of the Savannah region of Nigeria. Broadly, certain animal characteristics and poor husbandry management practices contributed to increased prevalence of SCM. While this study has certain limitations, such as non-independent sampling and spatial auto-correlation, an effort was made to ensure geographical spread and adequate representation of cow herds and farm types that exist in Sokoto State. The overall majority (over 90%) of the herds in Sokoto are semi-intensive or extensive herds. The purely extensive herds were not particularly selected in this study because lactating cows are usually resident under the semi-intensive system while other cows are taken out to pastures. We believe this sampled population represents the available herds in the region.

While we are aware that the testing system is somewhat subjective and may incorporate some misclassification/overestimation bias, we made all effort to reduce any bias by: a) allowing three technicians to conduct some preliminary tests on known samples and testing the kappa statistics for strength of agreement between the individual scores of the three testers (Kappa = 0.70 (0.40-0.99 at CI_95%_); b) usage of pasteurized milk samples as gold standard for negative test; c) carrying out random bacteria culture and analyses of selected milk sample to match them with the CMT scores [16]; and d) we are aware that convenient sampling of herds which was done may bias the outcomes of our investigation, however it is difficult to randomize the herds in view of frequent movement and change of locations most of this herds were subjected to. Finally, the manufacturer’s instructions for the use of the kit were adhered to in carrying out the test. Bias in body condition scoring was reduced by using the same standard (modification of ELANCO-Kellogg’s pictoral chart) in scoring all animals [17]. Interviewers’ and courtesy biases were reduced by allowing the farmers to give free opinions on all issues and by asking certain check questions in addition to questions needed to collect the required parameters.

An overall quarter-level prevalence of 43.25% of SCM was observed, based on CMT scores, and but the LFQ were more affected than the other quarters (Table 1). Though an immediate explanation cannot be established for this observation, it is highly likely that in the process of milking, these particular quarters were milked first before the other quarters because most of the operators tend to be right handed and sit first to the left of the animals. It is also possible that contaminations from the operators left hands, which are used for less hygienic purposes (sometimes without proper washing and disinfection), and which are most often used to milk the LFQ and the RHQ (based on sitting position) contributed to the higher incidence observed in these two quarters. At the herd-level, the prevalence was 85.33%. This value is higher that those obtained previously in Tanzania (43.25%), [18]. The fact that the prevalence of SCM was higher in a single quarter (32.33%) and reduced as more quarters are affected (30.33%, 16.00% and 6.67% in 2, 3 and 4 quarters respectively) is an indication that possibly, one quarter is usually first infected and the others become affected through contamination and other means especially during the milking procedures.

Among the breeds, the Rahaji and Sokoto Gudali were more frequently affected with documented prevalence of 65.91% and 45.67%, respectively (Table 2). The reason for the breed differences is not clear although these more affected breeds were primarily beef cattle, while Holstein-Friesian and White Fulani are better “milkers” and are preferentially selected by dairy farmers in Nigeria. It is also possible that environmental selection has caused some breeds to better adapt to a less hygienic environment, the main source of teat contamination and SCM. Such adaptations may include narrower teat canal or firmer sphincters at the tip of teats. The results from the multivariable model support this suggestion. Future research may critically evaluate breed differences to understand their individual contributions to SCM.

A statistical association was found between the four risk factors, age (in years), washing of hands before milking, breed of a cow, and different mammary quarters. Younger cows were less likely to be afflicted with SCM. This could be explained by the fact that the teat canal in older animals is more dilated or it remains partially open permanently due to years of repeated milking. This encourages the introduction of environmental and skin-associated microorganisms into the teat canal, leading to SCM and milk production losses. Schroeder had previously stated that milk production losses are nearly double for older cows than in first lactation cows [19].

Cows in herds where hand-washing before milking is being frequently practiced had a reduced risk of developing SCM compared to herds with less hygienic milking practices. Certain infectious organisms are normal residents of human hands and these microbes could be transmitted to uninfected animals and quarters during milking. The importance of thorough teat and hand washing before milking cannot be overemphasized in view of this finding. Where feasible, the use of hand gloves during milking should be encouraged. This has similarly been established in past reports [1,20]. Our survey found that approximately 37.33% of the farmers do not wash their hands before milking, and the value of hand washing is an important message that extension veterinarians can pass along to farmers to reduce the risk of SCM.

The level of SCM in lactating cows in this study is comparable or higher than those obtained elsewhere in Africa (Tanzania = 51.6-75.9%, [18,21]; Ethiopia = 80%, [22]), and it is indicative of poor management of animals and a heavily contaminated milking environment. Though this study is based only on the qualitative test of CMT, we believed that there is a need to conduct laboratory evaluations and establish the pathogens that may be involved in this observation. Such microbes may include *Escherichia coli* and *Streptococcus uberis* amongst others. There may also be a need to conduct broader studies, taking into account regional and socio-cultural differences, to determine the effect of other potential risk factors not included in this study, such as season and geographical location of herds.

## Conclusions

Since a sound mastitis control programme can only be designed with the full knowledge of environmental and potential risk factors, this work should assist dairy cattle veterinarians in planning and adopting preventive practices to decrease both the cow and herd-level incidence of SCM and improve milk quality.

## Methods

### Study area

Prior to the commencement of the study, project and ethical clearance was obtained from relevant authorities of the Faculty of Veterinary Medicine, Usmanu Danfodiyo University, Sokoto*. Owner’s consent was obtained at the time of sampling. This study was conducted among cow herds located in Sokoto State, Nigeria. Sokoto State is located in the extreme northwest region of Nigeria between longitudes 4° 8’E and 6° 54^’^E and latitudes 12° and 13° 58’N. It shares boundaries with Zamfara State to the East, Republic of Niger to the North and Kebbi State to the west and southwest [23]. It has a land area of about 32,000 km^2^ and a tropico-continental climate which is broadly grouped into two including the moist condition with peak annual rainfall of approximately 550 mm in August and a dry harsh condition which presents both as an initial cold Harmattan (dry North-easterly wind with dust) from October to March, and a hot dry season in April to May with temperature peaking at 100° F (39°C) and a humidity of less than 20% [23].

While a sizeable proportion of the cattle population in Sokoto are resident herds, an equally large population of animals are migratory (trans-humance, pastoral or trade). These animals cross the inter-state and national boundaries with implications for spreading diseases. Sokoto State ranked the second-largest in Nigeria in term of animal population with an estimated livestock population of about 2 million cattle, 3 million sheep, 5 million goats, 4,600 camels and variable species of poultry including chickens, guinea fowls, ducks and turkeys [24]. These animal species serve as the source of meat, milk, egg and other animal products to the inhabitants of the state. The cattle herds in Sokoto state vary widely in size from a few animals (usually ≤5) up to a few hundreds [25,26].

### Sample collection and California Mastitis Test

Three hundred cows were recruited from seventy-seven herds comprised of 9 intensive/sedentary (11.69%) and 68 semi-intensive/partially sedentary (88.31%) systems of management. The inclusion criteria were the willingness to participate in the study, agreement to allow access to all animals for the period of the study and presence of lactating cows in the herd. Though the herds were conveniently selected, selection of participating cows from each herd was done randomly. All intensive herds engaged in the study were on commercial farms. The survey was carried out between August and October 2011. The target population were lactating cows (n = 300) within the herds ranging from 1 to 110 cows (median = 6, min = 1, max = 110). These animals included 44 White Fulani (median = 5, min = 1, max = 110), 104 Sokoto Gudali (median = 5, min = 1, max = 110), 141 Holstein-Friesian (median = 52, min = 1, max = 110) and 11 Rahaji breeds (median = 5, min = 1, max = 6). CMT, an indirect measure of the health status of the udder, was conducted on a total of 1,200 (300 × 4 quarters) individual quarter milk samples obtained from 300 lactating cows. This test and its effectiveness in detecting SCM has been established and previously validated based on somatic cell counts (SCC) [27-31]. Prior to milk sample collection, udders and teats were cleaned with alcohol and dried in order to avoid presence of feacal debris in the milk as it could interfere with the interpretation of CMT result. Milk samples were collected directly from each quarter of the udder of a lactating cow into the corresponding segment of the four-quartered paddle. Quarters were identified as left fore-quarter (LFQ), right fore-quarter (RFQ), left hind-quarter (LHQ), and right hind-quarter (RHQ). The surplus milk was sucked out of the paddle leaving approximately 2 ml in each segment.

Two (2) ml of the CMT-test liquid was added into each segment of the paddle and mixed gently to test for SCM. The results for CMT were read in approximately 10 seconds and were recorded as – (negative; SCC score of ≤100,000 cells/ml), + (weak positive; SCC score of >100,000 – 500,000 cells/ml), ++ (positive; SCC score of >500,000 – 1,000,000 cells/ml), and +++ (strong positive; SCC score of ≥1,000,000 cells/ml), as described by the manufacturers (Schroeder, 2010). SCM has also been defined previously to include any mammary gland achieving a test result with a quarter somatic cell counts (QSCC) of >2 × 10^5^ cells/ml. A value of >100,000 cells/ml was used as the cut-off value for an intra-mammary infection for our diagnosis of SCM [32,33]. All CMT scores of 0 and trace (<100,000 cells) were considered as negative while CMT scores of +, ++, and +++ were considered indicators of SCM. The test mixture (milk sample and CMT-test liquid) was discarded and the paddle washed with clean water and dried properly after each use to enable the paddle to be used for another lactating cow in the same herd. For every herd, a new paddle was used.

### Epidemiologic questionnaire and herds surveyed

A closed-ended questionnaire (n = 77) modified from a study by Plozza *et. al.*[1] was administered to each farmer or cow handlers at the time of sampling to collect information about improving udder health. A pilot survey of 5 participating herds was conducted to validate the questionnaire and reduce ambiguity before the commencement of the main study. Questions relating to improving udder health of the herd were asked during each visit (Table 4). Data on animal characteristics were collected from the farm record or cow owner. These included: cow type/origin, age using dentition, and breed identification using specific skin markings and phenotypic characteristics. An assessment of body condition score (BCS) was done according to de Souza *et al.*[34].

### Statistical analysis

Data on animal characteristics and herd management practices, including the results of the individual QSCC, by cow and herd identifications, were recorded in the field form and transferred into MS-Excel® for Windows 2007 (Microsoft Corporation, Redmond, USA). Data from all questionnaires were verified, rechecked and filtered by three persons. Variables with many outliers and wide disparities were dropped to prevent bias in the analyses. The “age” variable (mean = 6.04 years, SD = 1.97, min = 3, max = 14) was split into 2 categories [young or cows that are ≤5 years old (n = 142, mean = 4.46 years, min = 3, max = 5), and old that are > 5 years (n = 158, mean = 7.45 years, min =6, max = 14)]. For the purpose of building the logistic regression model, a quarter was defined as CMT positive if it had a CMT score of 1+ or above. A lactating cow was defined as CMT positive if it had at least one quarter with a CMT score of 1+ or above, hence, all CMT scores of negative (−) were coded as 0 and all positive scores of +, ++, +++ were coded as 1. Similarly, all of the explanatory variables were coded appropriately before the data were transferred into Stata® v. 10 (StataCorp, College Station, Texas 77845 USA) for statistical analyses.

Based on the results of the CMT, the prevalence of SCM by quarter and cow breed was calculated. The effects of herd management and animal predictor covariates on the probability of QSCM were initially investigated using a univariable analysis. Only variables that had unconditional association with the outcome that were significant at a probability value *P* ≤ 0.20 in the univariable analysis were retained for further investigation in the multivariable logistic regression base model [35], with cow nested in herd being random factors. The correlations between the selected explanatory variables were analyzed for each category of herd management and animal characteristics. In the logistic model, CMT score (1/0) was the dependent variable and cow and herd were incorporated as random (frailty) effect term to address potential data clustering at cow and herd levels. A Likelihood ratio test (LRT) was performed via stepwise backward elimination procedure. Reduced models were compared with full models in order to assess whether the new model was a better “fit” than the other candidate models. All statistically significant variables (*P* ≤ 0.05) were kept in the model based on the LRT. Confounding was assessed every time a non-significant variable was dropped from the model by comparing the change in the ORs for the variable remaining in the model. Any variable that caused a 20% or greater change to the ORs of a statistically significant variable when removed from the model was considered as a potential confounder [35].

### Ethical approval

*At the time of carrying out this project, no standing Committee existed specifically for ethical approval of projects. However, ethical clearance was part of the discussion and approval process of project proposals by the Large Animal Unit, Usmanu Danfodiyo University, Sokoto, Nigeria (August, 2011).

## Competing interests

We declared no competing interest.

## Authors’ contributions

AS designed the study; AS and JA supplied the reagents and collected samples; AAM coordinated the research; AS and FOF modeled data, conducted statistics and interpreted data; all authors (AS, JA, AJ, AAM and FOF) participated in manuscript drafting and final approval. All authors read and approved the final manuscript.
